# Executive summary of AAPM Report Task Group 113: Guidance for the physics aspects of clinical trials

**DOI:** 10.1002/acm2.12384

**Published:** 2018-06-29

**Authors:** Jean M. Moran, Andrea Molineu, Jon J. Kruse, Mark Oldham, Robert Jeraj, James M. Galvin, Jatinder R. Palta, Arthur J. Olch

**Affiliations:** ^1^ University of Michigan Ann Arbor MI USA; ^2^ University of Texas MD Anderson Cancer Center Houston TX USA; ^3^ Mayo Clinic Rochester MN USA; ^4^ Duke University Medical Center Durham NC USA; ^5^ University of Wisconsin Madison WI USA; ^6^ Imaging and Radiation Oncology Core Philadelphia PA USA; ^7^ Virginia Commonwealth University Richmond VA USA; ^8^ University of Southern California and Children's Hospital of Los Angeles Los Angeles CA USA

**Keywords:** clinical trials, external beam, protocols, quality assurance, standardization

## Abstract

The charge of AAPM Task Group 113 is to provide guidance for the physics aspects of clinical trials to minimize variability in planning and dose delivery for external beam trials involving photons and electrons. Several studies have demonstrated the importance of protocol compliance on patient outcome. Minimizing variability for treatments at different centers improves the quality and efficiency of clinical trials. Attention is focused on areas where variability can be minimized through standardization of protocols and processes through all aspects of clinical trials. Recommendations are presented for clinical trial designers, physicists supporting clinical trials at their individual clinics, quality assurance centers, and manufacturers.

## 1 ABOUT THIS EXECUTIVE SUMMARY

The full report of AAPM Task Group 113 on Guidance for the Physics Aspects of Clinical Trials is available at the AAPM Reports website. This executive summary provides an overview of the major headings of the full report. In addition, details were retained in this report to highlight a few areas where there has been an evolution in clinical trials. Appendices A–D include all of the TG113 recommendations with the reference information contained in the full report.

## 2 INTRODUCTION AND CHARGE OF THE REPORT

There is growing evidence^1^, ^2^, ^3^, ^4^, ^5^ on the need for standardization of treatment planning and delivery methods to ensure quality in clinical trials to help support the investigation of new safe and effective treatments and/or assessment methods in multi‐institutional settings. Such standardization will improve the consistency of the radiotherapy received by patients and the radiotherapy data submitted for a given clinical trial. These data are required to validate that all patients in each arm of a given study received the therapy as intended. Violating this assumption can jeopardize the validity of the outcomes reported by the trial group.

**Figure 1:**
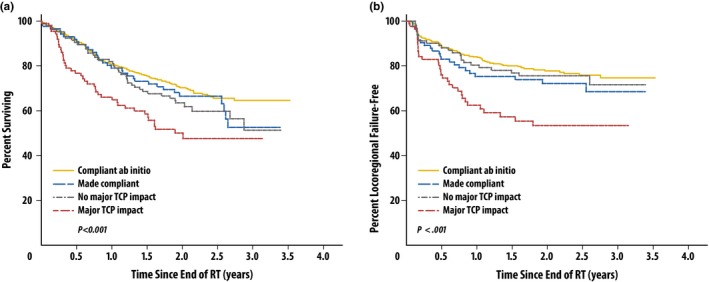
Peters et al. assessed the impact of protocol compliance for TROG 02.02 on advanced head and neck cancer and demonstrated an impact on a) overall survival and b) time to locoregional failure as a function of the deviation status. (Figures 2 and 3 reprinted with permission from Peters et al., JCO, 28: p. 2999.)

A related consideration that affects overall quality is the ability of those participating in clinical trials to create plans as part of their standard clinical flow that are both compliant with protocol specifications and optimal. The importance of compliance in trials and the impact on detecting changes in outcome have been demonstrated in a number of trials,^1^, ^2^, ^3^, ^4^, ^6^ such as TROG 02.02 on advanced head and neck cancer (Fig. 1), and in meta‐analyses of other trials. When designing a trial, the planning guidelines are set to be able to answer the clinical trial questions. However, there may be variation in planning methods, and a planner may not know when a better (such as improved target coverage with reduced dose to normal tissues) plan is reasonably achievable without real‐time feedback during the planning process. Knowledge‐based planning, where the achievable dose‐volume metrics from previous patients can used to predict each new patient's DVH, was shown to retrospectively identify plans which were clinically acceptable but suboptimal in the context of the clinical trial.^7^ For example, plan quality was analyzed for patients treated on RTOG 0126 exploring the relationship between plan quality and rectal toxicity. Suboptimal plans were identified by comparing predictions for target and organ‐at‐risk doses to those that were submitted as part of a trial for 219 IMRT patients. The library was created from plans which were defined as the best from the protocol based on a risk evaluation. This work highlights the challenge of using a series of DVH points alone as the primary guidance to create a treatment plan. There is a richness of information available when comparing a new plan against a library of plans that have been previously determined to be optimal and protocol compliant. Improved planning tools such as those with knowledge‐based planning have been needed for some time to provide detailed feedback to institutions on whether or not their treatment plans not only meet the dose‐volume histogram requirements but are also optimal for use in clinical trials. With respect to quality assurance requirements, there are important ongoing efforts toward global harmonization of quality assurance^6^ (such as structure nomenclature addressed by AAPM Task Group (TG) 263^8^) for radiation therapy clinical trials.

The charge of AAPM TG 113 is to:

1. recommend physics practices for clinical trials involving external photon and electron beam radiation therapy that ensure minimum standards for data quality in clinical trials.
2. identify opportunities to improve consistency in each part of the planning and delivery process.
3. provide guidance to QA organizations on how best to support the spectrum of radiotherapy clinical trials, from those with basic to advanced technology.
4. provide suggestions regarding the credentialing requirements to reduce potential inconsistencies in the radiotherapy process.

The use of protons or brachytherapy in clinical trials is outside of the scope of this document. Throughout the report, recommendations are presented in each section for major areas of the process from simulation through treatment delivery in the context of clinical trials. The recommendations are organized by the categories of clinical trial designers, physicists (at the local institution), quality assurance (QA) centers, manufacturers, and advanced technology trials and are also presented by category in Appendices A–D. The full report includes information on restructuring of the clinical trials network and associated QA centers funded by the NCI.

## 3 THE ROLE OF THE PHYSICIST IN CLINICAL TRIALS

Physicists play different roles with respect to clinical trials. At institutional, national, and international levels, physicists may be lead or co‐investigators representing clinical and technical components. In the context of clinical trial groups, physicists may lead or co‐design a clinical trial. For national trials supported at individual institutions, physicists play a key role with physicians in ensuring protocol compliance. Other perspectives include physicist roles in QA centers and as employees of a manufacturer whose products are being used to support clinical trials.

**Figure 2:**
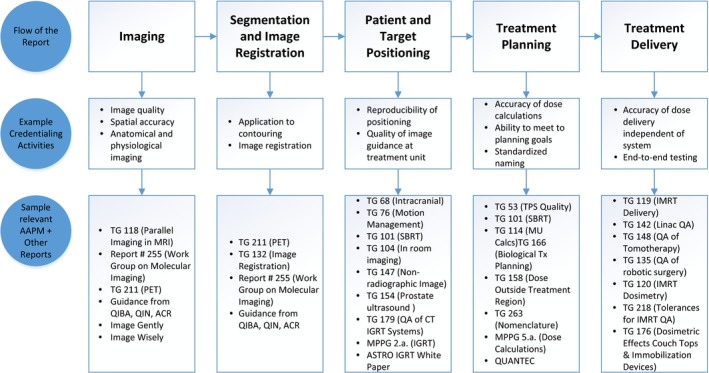
This diagram shows the flow of the AAPM TG 113 report with respect to the patient treatment process (top row). Example credentialing activities related to quality in clinical trials are shown in the middle row. The bottom row shows AAPM and other reports that are related to the areas in the top row.

TG 113 considers the entire process designing a trial and its QA through the activities of the local team from simulation to planning and treatment delivery to improve the consistency for clinical trials, whether trials are funded by NCI, industry, or other entities. Many AAPM task group reports are relevant to the work of TG 113. Figure 2 shows an overview of the major areas involved once a patient is enrolled in a clinical trial. For each area, both sample relevant task group reports and credentialing types are noted. Many of the referenced task group reports are ones that are already relevant to the practice of clinical medical physics in radiation therapy which then have an impact on the treatment of patients enrolled in clinical trials. Therefore, minimal additional references are made to task group reports throughout this report.

## 4 IMAGING

Image quality is paramount to many clinical trials for both target definition and treatment assessment. This section makes recommendations to facilitate consistent and accurate volume definition for clinical trials. Numerous collaborative efforts are focused on standardization of imaging, including quantitative applications. Formed in 2008, the Quantitative Imaging Biomarkers Alliance (QIBA) involves drug and equipment companies and imaging societies and has a charge to develop and advance standards for the use of volumetric computerized tomography (CT), positron emission tomography (PET), and magnetic resonance imaging (MRI) in clinical trials. QIBA has created validated datasets including ones that can be used for evaluating lung nodules^9^ and phantom datasets that are used to validate analytical tools such as dynamic contrast enhanced MRI.^10^ The Uniform Protocols for Imaging in Clinical Trials (UPICT) initiative has created a protocol for trials involving imaging with FDG‐PET/CT.^11^ Several groups within the AAPM are actively advancing the use of quantitative imaging information, and guidance will continue to evolve in this area.

Some clinical trials require credentialing or a central imaging review by QA centers that have expertise in quantitative imaging, such as IROC Ohio, IROC Philadelphia (DI), and IROC Rhode Island. Credentialing may evaluate characteristics, such as image quality, spatial integrity, and contrast; the requested characteristics depending on the role of imaging within a given trial. For example, considerations with respect to understanding uncertainties in molecular imaging have been described.^12^ More details regarding quantitative imaging in clinical trials are presented in the full report.

## 5 SEGMENTATION

Accurate segmentation is a critical task in clinical trials. Important technical sources of variation in segmentation include variable window and level settings, the use and sensitivity of auto‐segmentation algorithms to input parameters, and inappropriate margin expansion algorithms. For example, inappropriate window and level parameters can lead to significant bias and errors in volume definition with one study identifying factors leading to variations up to 42% by clinician which were reduced by using a standard protocol.^13^ Improvements in the consistency of contours are seen when pretreatment reviews of contoured structures are performed by protocol principal investigators. Training, such as via workshops or webinars, should be provided to physicians and other personnel for a given trial if there could be significant variability in the delineation of structures.

For organs which will be evaluated with dose‐volume histograms (DVHs), the protocol should specify how much of the organ must be contoured. For example, it may be appropriate to specify a region of spinal cord to be contoured with respect to the superior and inferior borders of the PTV. Structures with mean dose objectives should be contoured in their entirety. For structures where the entirety may not be included within the planning scan, the protocol should specify dose limits in absolute (cc) instead of relative (%)volume.

It is crucial that protocol designers provide explicit guidance in how structures are defined, especially when multiple structures are involved. Significant differences have been shown in dosimetric parameters for lung cancer for different definitions of normal lung, the gross tumor volume, clinical target volume, internal target, or planning target volume.^14^ Variability of such definitions in a clinical trial would have a significantly detrimental impact on the ability of the trial to resolve the study question. It may also lead to inconsistency in the application of dose goals if the same dose goals are used but with different definitions from one trial to another. Therefore, definitions and dose goals across trials to the same body site should be standardized as much as possible with the expectation of evolution of care over time. In addition, the protocol should specify any additional limits to doses to organs outside the treatment field.^15^ A final critical concern is that some systems ignore the volume of an organ outside the dose calculation grid when reporting dose‐volume parameters. For such systems, the dose‐grid should cover the entire organ of interest so that derived dose‐volume parameters used for treatment planning represent the entire organ. Additional details and recommendations regarding segmentation are found in the full report.

## 6 IMAGE REGISTRATION

Clinical studies that require multiple image datasets need to use image registration software. When multiple image modalities are used for treatment planning, the protocol designers should consider providing specific recommendations for internal or external landmarks that can validate the adequacy of the registration for treatment planning.

If the accuracy of the image registration for each patient affects the quality of the trial (such as in defining the target volume), the protocol designers and QA centers should require credentialing of the image registration software by using phantoms of known geometry and should follow the guidance of AAPM TG 132.^16^ The physician directive should specify the goals of the image registration, the method and what anatomical region should be emphasized in the registration.^16^

With respect to how image registration is used at the treatment unit, the trials designers should determine if it is necessary to distinguish between applications for target and normal tissue definition compared with daily online treatment guidance. Image registration considerations, which are described in the full report, may also differ if there is a midcourse plan adaptation and dose accumulation methods are utilized.^17^

## 7 PATIENT AND TARGET POSITIONING

Patient and target positioning is affected by immobilization and the frequency and type of image guidance used at the treatment unit. The margins for treatment planning are affected, as well as the achievable accuracy of image registration using multimodality imaging scans which are used to design and assess patient treatments, especially dose–response studies for clinical trials.

In the context of clinical trials, the type of recommended immobilization described and/or required in a particular trial depends on (a) the available and acceptable equipment in potentially accruing clinics, (b) the accuracy required by the protocol; and (c) the frequency and accuracy of the treatment guidance methods that may be recommended during patient treatment. Trial designers should determine if a given trial requires specific immobilization, such as for stereotactic radiosurgery or stereotactic body radiation therapy. More details regarding immobilization considerations are available in the full report.

Protocols should be specific with respect to the type and frequency of image guidance. The relationship between localization methods and the appropriate PTV margin^18^ should be considered in the design of all clinical trials. For example, a trial involving treatment of breast cancer may involve weekly portal imaging, whereas a trial involving SBRT may require daily volumetric imaging. As described in the full report, the designers of clinical trials should be specific with respect to the recommendations for intra‐ and inter‐treatment margins in a given trial for consistency and reproducibility.

## 8 MOTION ASSESSMENT AND MANAGEMENT

For many treatment sites, physiological motion must be assessed to determine if management of that motion is necessary for segmentation and treatment delivery. The AAPM Task Group 76 report, published in 2006, provides guidance for considerations at simulation and for treatment planning.^19^ Efforts are under way to update that report with guidance needed today for clinic care and clinical trials. In 2017, several members of the Medical Physics Committee of NRG Oncology reviewed guidance in the context of stereotactic body radiation therapy for thoracic and upper abdominal tumors and made recommendations in the context of clinical trials.^20^ They describe considerations regarding both motion assessment and motion management.^20^ The full report of TG113 contains further discussion of these considerations.

## 9 TREATMENT PLANNING CONSIDERATIONS

With respect to treatment planning, there are considerations related to the treatment planning system itself as well as the creation of treatment plans for a given clinical trial. For example, more accurate model‐based algorithms rather than pencil beam algorithms should be used for planning for patients in clinical trials. Recommendations are also provided in the full report for clinical trial designers and physicists at local institutions emphasizing tools that support improved quality for clinical trials and that may improve efficiency as well.

Protocol designers and manufacturers may be able to provide templates and tools that can be used to support the uniform implementation of clinical trial guidelines. These tools may include structure templates that work on multiple vendor platforms such as following the nomenclature recommendations of AAPM TG 263 and advanced planning tools that aid in meeting the dosimetric requirements of a protocol. For example, a dosimetric model could be developed for knowledge‐based planning or a script could be created with standard input such as the beam energy, beam arrangement, and modality to best meet a given protocol.

**Figure 3:**
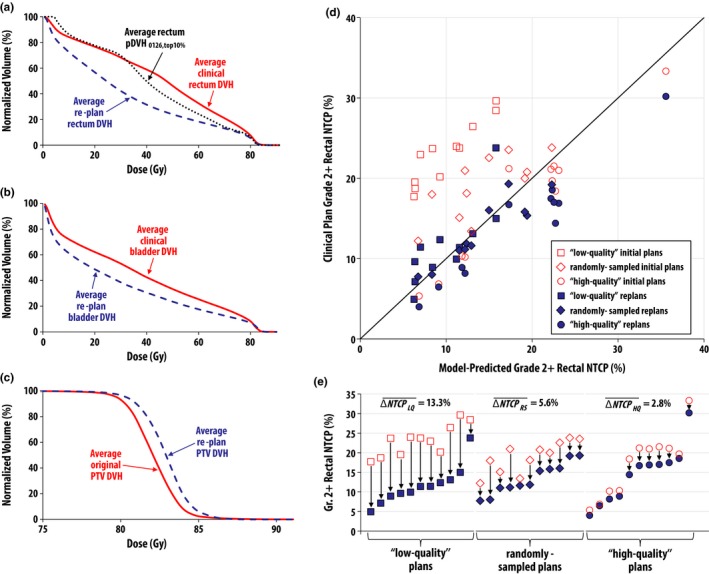
Moore et al. retrospectively evaluated the impact of knowledge‐based methods, such as calculation of a predicted DVH (pDVH), on the overall IMRT plan quality for RTOG 0126 for prostate cancer and the resulting predicted grade 2 rectal normal tissue complication probability (NTCP). (Figure courtesy of Moore et al.^7^).

Advances are being made in the use of automated tools for planning and for assessing the consistency of a treatment plan with respect to previous clinical trials. This development has important implications for clinical trials both for secondary analyses and for more robustly assessing plan quality during the accrual phase of a trial. The ability to improve plan quality using knowledge‐based methods was evaluated for RTOG 0126 where predictive DVHs showed that further sparing of normal tissues was achievable with a group of plans (Figure 3).^7^ Figure 3(e) demonstrates that plans which were defined as “low quality” had significant improvements with respect to the predicted rectal toxicity based on the calculated normal tissue complicated probability values for each plan. Such tools will be valuable both for the teams at the institution performing treatment planning for protocol patients as well as for the analysis of plan quality at the QA centers (https://www.nrgoncology.org/Scientific-Program/Center-for-Innovation-in-Radiation-Oncology).

Additional considerations include considerations specific to adaptive therapy and re‐irradiation. Emerging new technologies in radiation treatment planning and image guidance will place additional requirements on the capabilities of the TPS. Investigators and manufacturers are developing tools to better support adaptive therapy such as deformable image registration and the creation of a model based on the accumulated dose to a patient.^21^ Many of these considerations are beneficial for patients who are retreated which may also be a component of a clinical trial. Deformable registration and fusion algorithms are currently being investigated and should ultimately be included in the software tool set available at individual institutions and at QA centers. These algorithms are an integral part of accurately assessing and reporting the dose given to the patient throughout the course of therapy. To fully appreciate the impact of anatomical changes for case review in a clinical trial, the composite delivered dose would be best, but if not available, multiple imaging studies, their time sequences, and all treatment plans should be submitted to the QA center.

## 10 TREATMENT DELIVERY DOCUMENTATION

Treatment management systems permit verification that the correct energy, beam modifiers, monitor units, treatment dates, and number of fractions were used for individual patient treatments. A summary of this information should be exportable in a standard format for a clinical trial. This information is crucial because it has been shown that some patients may have poorer outcomes as a result of missed radiation therapy treatments.^22^ Missed treatments may also impact the interpretation of the effectiveness of a clinical trial if not documented and considered. Clinical trial groups should consider the implications of missed treatments and how best to collect the information.

## 11 QA CORE FUNCTIONS AND INSTITUTIONAL PREPARATION

Credentialing for clinical trials is the performance and documentation of specific processes by an institution and its team to demonstrate their ability to accurately plan and treat patients for a particular protocol or treatment modality. In addition, a part of credentialing verifies that the institution is capable of submitting the required datasets to the QA center. The credentialing process is designed to ensure that all participating institutions can faithfully apply the protocol guidelines and deliver comparable doses in a clinical trial. This improves the ability to detect outcome differences within a given trial.

Clinical trial groups face a challenge in determining the safest way to adopt and incorporate new technologies in both existing and newly developed clinical trials. When incorporating new or less uniformly applied technologies in clinical trials, the results of credentialing tests aid in discovering and correcting variable, outlier, or noncompliant performance by participating institutions, and this helps to lessen the variability in protocol performance across all institutions. The test consist of a combination of questionnaires, benchmark plans, dry‐run digital data submissions, and phantom irradiations. If the institution passes the test, then it is approved for enrollment of patients for the pertinent protocol and the specified treatment modality. The full report has details regarding the purpose and types of benchmarks, credentialing techniques, phantom considerations, pretreatment, and on‐treatment review.

A kick‐off meeting is recommended with the appropriate research staff, clinical trials coordinator, principal investigator, physicist, dosimetrist, and a therapist before patients are enrolled on the protocol. Examples of the types of things to discuss at a kick‐off meeting are included in the full report.

## 12 SUMMARY

It has been shown that the quality and consistency of the trial impacts patient outcomes.^1^, ^2^, ^3^, ^4^, ^5^ This report identifies physics and other team member practices that specifically improve the treatment planning and delivery data for clinical trials. It provides benchmark and other quality assurance recommendations for groups which design and conduct clinical trials to minimize inconsistencies in the radiotherapy processes and treatment. The details for each major section along with recommendations are provided in the full report. The recommendations for the full report are presented in the appendices for the clinical trial designers (Appendix A), physicists at individual institutions (Appendix B), QA centers (Appendix C), and manufacturers (Appendix D).

There are unique challenges posed by advanced technology trials in a multi‐institutional setting. To achieve the desired level of statistical power in a clinical trial, the QA center must verify that the technology is implemented uniformly in multiple settings. The QA centers have had to adapt quickly as new technology becomes available and is implemented into clinical practice. Other guidance will need to be developed as current advanced technologies mature and other technologies develop.

With technological advancements, manufacturers play a role in the development of improved technology and in providing updates to software tools to enhance the conduct of clinical trials. Important work has been ongoing in harmonization of credentialing for clinical trials which the NCI has advocated along with other changes.^23^ Quality for NCI‐funded clinical trials continues to be supported by the IROC infrastructure. Finally, successful clinical trials involve a partnership relationship among all of those involved.^24^ Improved consistency in the design and performance of the physics aspects of clinical trials will help ensure that the data are of high integrity and can be used to answer the clinical trial questions and ultimately affect clinical practice.

## APPENDICES 1

### 1.1

These appendices consolidate the recommendations in the report for ease of access by clinical trial designers, physicists, QA centers, and manufacturers.

### APPENDIX A. RECOMMENDATIONS FOR CLINICAL TRIAL DESIGNERS

#### Imaging

- Determine if imaging‐specific credentialing is required through a review by imaging experts (such as the imaging organizations within IROC) and whether or not variability in techniques and/or variations in commercial scanner technology need to be considered.
- Design a standard operating procedure for imaging, incorporating expertise of imaging physicists/scientists where appropriate.
  - Specify the extent of anatomy to be imaged, including whole organs when required for dose volume analyses
  - Specify any timing requirements of the acquisition in relation to treatment start for all imaging data for treatment planning and assessment. Be explicit regarding patient preparations for imaging.
  - Keep image acquisition, reconstruction and analysis procedures consistent when multiple imaging sessions for a patient are required.
  - Ensure consistent patient set‐up and immobilization between different imaging modalities and treatment (see Sections 7 and 8) through credentialing of multi‐modality image registration.
  - Specify which contrast agents are permitted and provide details on the timing and amount of the agent to be used.
  - Provide guidelines on basic imaging parameters for trials permitting different modalities such as MRI, MRS, and/or PET/CT to account for the variability of different scanners.
  - Develop imaging benchmarks when modalities such as PET and MRS are used to ensure that the department's systems for contouring are capable of representing that data adequately in support of the clinical trial.

#### Segmentation

- Specify window and level values, when appropriate, for consistent visualization and segmentation.
- Refer investigators to published consensus atlases for target and organ at risk delineation as a reference when appropriate.
- Provide training to physicians for a given trial if there could be significant variability in the delineation of structures among physicians.
- Provide guidelines to physicians, physicists, and dosimetrists on how to address imaging artifacts that interfere with target or normal tissue segmentation (e.g. scatter from metal or the presence of contrast on a CT simulation scan).
- For organs which will be evaluated with DVHs, the protocol should specify how much of the organ must be contoured for structures such as the spinal cord.

#### Image registration

- For any applications of image registration in a trial, the protocol designers should specify which methods are allowed (rigid only, deformable), and any additional constraints.
- Guidance should be provided about how the quality of an image registration is judged which should distinguish between applications for target and normal tissue definition compared to daily online treatment guidance. This information should be considered when image registrations are evaluated as part of credentialing for a given trial.

#### Patient and target positioning

- The clinical trial designers should survey the literature including relevant AAPM Task Group reports to determine the type of immobilization suitable to meet aims of the clinical trial.
- Consult with physicist(s) at a lead institution and other possible participating institutions to ensure that the proposed accuracy limits are achievable at a number of centers.
- Clearly specify which immobilization equipment is required for the trial (where a preliminary assessment of equipment availability in the community could be done via the IROC Houston facility questionnaire if needed) or if certain types of equipment are not permitted.
- Use the most up‐to‐date terminology to specify definitions of target volumes in the trial design (e.g. ICRU #83 at time of publication).
- Review data in the literature to define acceptable PTV margins related to the technology used for simulation (such as 4DCT) and the frequency and type of imaging for the anatomical site.
- Provide explicit guidance on the contouring of targets and necessary expansions.
- If a protocol requires an evaluation of target margins midtreatment, the clinical trial designers should specify the frequency and methods of evaluation in the clinical trial design. For example, how to address changes in tumor physiology and/or shape such as changes to targets in the lung or head and neck region due to shrinkage or growth of the tumor.

#### Motion assessment and management

- For relevant body sites, specify that the degree of target motion should be assessed at the time of simulation. For treatment sites where the impact of motion can be crucial, it is recommended that QA centers develop guidance, with respect to the acceptable imaging techniques to assess motion, documentation of that motion for a given patient, and how the information should be incorporated for creating target volumes.
- Incorporate guidance on motion management techniques in which the range of motion is greater than published limits (or significant normal tissue sparing can be achieved through their use). For trials when target motion may be ≥5 mm and delivery of a high daily dose (e.g. SBRT), institutions should be required to document the assessment and follow formal guidance such as that provided by AAPM TG 76^19^ or other organizations such as NRG^20^ to ensure motion assessment and management information is accurately captured for patients enrolled on the trial.
- For protocols involving monitoring of intra‐fraction motion, provide information regarding the acceptable technologies for monitoring and the thresholds for evaluation. Information should be provided as to whether intra‐fraction monitoring is required and the acceptable methods.

#### Treatment planning considerations

- Specify standard structure names that must be used for the clinical trial (follow consensus guidance when available) such as provided by AAPM TG 263 or other appropriate ontologies.
- Use published information on normal tissue limits such as through consensus efforts as appropriate when specifying the limits to normal tissues.
- For organs which will be evaluated with DVHs, the protocol should specify how much of the organ must be contoured for structures such as the spinal cord.
- Specify spatial resolution requirements for dose and DVH calculations that are commensurate with target and organ‐at‐risk (OAR) sizes.
- Specify the use of 3‐D treatment planning for all clinical trials (excluding special procedures such as total body irradiation or total skin electron treatments).
- Require the use of more accurate algorithms (such as convolution/superposition, Monte Carlo) for trials where tissue heterogeneities may be significant.
- Develop credentialing approaches for new applications of the TPS, such as biological treatment planning.^25^ Credentialing may include intercomparison of results using standardized datasets.
- Specify the dose‐volume constraints for organs‐at‐risk and consider any special concerns such as the buildup region or structures outside the treatment area.
- Specify the minimization of the integral dose or total dose to other normal tissues that may not be contoured in trials which allow the use of dose optimization techniques.

#### Treatment planning delivery documentation

- Determine which aspects of the treatment history should be required as part of the data submission
- Require a record of missed treatments as part of the data submission.

#### QA core functions and institutional preparation

- For credentialing, explicitly state which structures must be delineated by a physician rather than other personnel.
- Work with QA center staff to determine the type of credentialing and if existing benchmarks or other credentialing tests are appropriate before designing new tests.
- Require a credentialing process with pre‐ or on‐treatment review for at least the first few cases and perhaps for all cases prior to treatment for trials that are dependent on consistent contouring of target and normal structures, adherence to strict margin expansions, dose‐volume constraints, and novel treatment techniques.
- Require credentialing of technologies which may be susceptible to significant inter‐institutional variability.
- Confirm with physicist stakeholders (such as the NRG Medical Physics group and the AAPM Work Group on Clinical Trials), physicians and administrators when necessary to assess there are enough centers with adequate equipment and personnel available to meet the specifications, guidelines, benchmarks, and credentialing requirements (by the center) in a timely manner [estimate time of needed training(s)].
- Require QA centers to confirm that the submitted treatment plan of a benchmark irradiation meets the specified requirements for the phantom plan, not only that the measurements and calculations are in agreement.
- For applicable treatment sites, require a benchmark test that assesses the accuracy of image fusion, IGRT, or other methods critical to the outcome of the trial performed by the institutional personnel routinely planning and treating patients in the clinical trial.
- The protocol should specify who reviews the case (QA center staff, study principal investigators and co‐investigators, or other designated reviewers), the number of cases from each center to be reviewed (e.g. the first 2 patients enrolled from a given center or based on compliance), the type and timing of the review, and whether or not the credentialing should be for each participating physician or the institution as a whole.

### APPENDIX B. PHYSICISTS AT THE LOCAL INSTITUTION

#### Imaging

- Train and work with the appropriate personnel to implement the protocol‐specified imaging standard operating procedures for image acquisition, reconstruction, processing, and analysis.
- Review patient imaging scans regularly to ensure compliance to the standard operating procedure.
- Consider the utilization of immobilization and setup methods and devices that are compatible with all imaging modalities used in the trial to reproduce the setup for the treatment planning CT.

#### Image Registration

- Evaluate the ability of the institution to follow protocol guidelines for segmentation and image registration.
- Follow recommendations of AAPM TG 132 with respect to image registration.^16^
- Adjust monitors for adequate resolution and properly calibrate for contrast and brightness to ensure consistency in target delineation.^26^ Note minimum settings in the standard operating procedure.

#### Patient and target positioning

- Determine that the institution's immobilization equipment is appropriate for the clinical trial before IRB submission.
- Ensure consistency of equipment for planning and treatment, for example, flat table tops for diagnostic scanners, use of compatible immobilization equipment for imaging scans when possible.
- Confirm the accuracy of the immobilization method used in the clinic for the protocol.
- Ensure personnel are adequately trained to support the process.
- For each protocol, understand how target margins are specified and make sure the margins are reasonable for the department's imaging, immobilization, planning, delivery, and treatment guidance process for the patients enrolled on the trial.
- For each protocol, monitor the effectiveness of the patient localization method for the patients enrolled on the trial.

#### Motion assessment and management

- Confirm that the motion assessment and management guidance specified in the protocol is followed whenever the range of motion meets published guidance limits.
- Ensure that the contoured IGTV is reasonable considering the measured motion for a given protocol patient.

#### Treatment planning considerations

- Ensure that the TPS is capable of meeting protocol requirements by:
  - Use of a model‐based algorithm such as convolution/superposition, Monte Carlo, or deterministic methods
  - Accurate modeling of beams and output factors, especially for small fields and IMRT techniques
  - Validating the dose‐volume histogram and analysis algorithms
- Ensure that 3D volumetric information can be exported to the Clinical Trial QA Center in DICOM‐RT format.
- Implement templates in your treatment planning system to use the standard names for targets and structures as specified by the clinical trial designers.
- Coordinate an end‐to‐end dry run of the protocol at his or her center on one of their patient dataset(s). (Note that this requires support from the department's administration for this valuable effort.)
- Determine the degree of attenuation by immobilization equipment and determine whether the attenuation should be accounted for in monitor units (MU) calculations.

#### QA core functions and institutional preparation

- Repeat the credentialing benchmark if a major change is made that may affect the quality in the clinical trial. Changes such as to the dose calculation algorithm may only require a resubmission of calculation data results rather than a re‐irradiation.
- Read the protocol and become familiar with the protocol guidelines and credentialing requirements to serve as the institutional expert on the planning and delivery details of each protocol that involves radiotherapy.
- Complete the Credentialing Status Inquiry (CSI) form and request the credentialing phantom for a particular trial, if needed. Treat the phantom as a patient, including involvement of the appropriate personnel. Return the phantom to the QA center in a timely manner.
- Work with the institutional team, including the physician, to ensure a kick‐off meeting for the protocol and to create protocol‐specific simulation and planning directives to ensure protocol compliance.
- Coordinate, develop, and perform an end‐to‐end test for a given protocol where each team member does his or her part to test drive and make corrections to the process before the first protocol patient is enrolled.

### APPENDIX C. RECOMMENDATIONS FOR QA CENTERS

#### Imaging

- Specify if an existing imaging benchmark would be beneficial for ensuring that enrolling institutions would be able to acquire scans of the appropriate quality to support the trial.

#### Image registration

- Develop imaging benchmarks as needed including when modalities such as PET and MRS are used to ensure that the department's systems for contouring are capable of representing that data adequately in support of the clinical trial.
- Develop credentialing methods incorporating deformable image registration following the recommendations of AAPM TG 132.^16^

#### Patient and target positioning

- Confirm that the precision of commercial immobilization systems and field experiences indicate that the proposed techniques realistically can meet the accuracy requested in the protocol.
- Ensure the appropriateness of the margin for a given trial.
- Determine credentialing methods for new techniques such as those requiring intra‐fraction monitoring.

#### Motion assessment and management

- Determine if a motion benchmark is required in support of specific trials with motion considerations using existing benchmarks where reasonable.

#### Treatment planning considerations

- Enable as much automation of data submission as possible.
- Continue validation and cross‐comparison of the performance of different dose algorithms with other QA centers and revise requirements as appropriate.
- Work with manufacturers to design interfaces that can be customized for electronic submission of all necessary protocol data.
- Provide the clinical trial groups with a template of standard target and structure names so that the clinical trial designers use consistent names across clinical protocols. Once available, the nomenclature of AAPM TG 263 should be followed.
- Develop mechanisms to share scripts or other tools (such as Excel Sheets with Macros enabled) to aid the institutional teams in assessing whether or not protocol guidelines are met prior to submission to the QA center. Tools could potentially be developed on multiple TPS platforms.

#### QA core functions and institutional preparation

- Regarding data format:
  - Have a methodology for anonymization of patient data if appropriate for a benchmark planning study. For example, TRIAD (NRG Oncology) includes an anonymization function.
  - When needed for a study, image format should be DICOM or DICOM RT (as appropriate) for CT, MR, PET, portal, simulator, and DRR images.
  - When needed for a study, structure set, plan and dose files should be in DICOM RT format.
  - Supplemental data that needs to be submitted to QA centers should be able to be electronically submitted.
- For new protocols, determine if an existing benchmark would meet the testing needs of the clinical trial.
- Develop benchmarks which are applicable across cooperative groups.
- Annually review facility questionnaires for all institutions participating in clinical trials.
- Determine when re‐credentialing is necessary.
- Provide appropriate benchmark phantoms for each trial that requires them, as resources permit.Existing phantoms should be assessed for suitability before new ones are made.
- Determine benchmark acceptability based on reasonable clinical practice for the radiation treatment convolved with the 90% confidence limit of the dose measurements by the QA center.
- Make information available to team members at an institution to determine eligibility for a given trial based on past credentialing efforts.
- When new planning and delivery techniques are introduced, evaluate the consistency with a subset of centers. This information should aid in assessing the appropriateness and need of a phantom irradiation.
- When large variability exists in benchmark results, work with key stakeholders to identify causes and methods to minimize dosimetric discrepancies. This may include working with physicists at local institutions as well as with manufacturer representatives.
- Develop with imaging experts a suite of benchmark phantoms and a robust program for image acquisition QA with different systems.

### APPENDIX D. RECOMMENDATIONS FOR MANUFACTURERS

#### Imaging

- For a given registration, develop methods to capture the primary goals of the image registration (e.g. target evaluation or organ‐at‐risk) and the goodness of the registration (see TG132 recommendations)^16^
- In image registration software, provide the ability to export necessary data for QA centers to be able to assess the quality of a registration (quantitative and qualitative) and export the needed information for straightforward review by those credentialing for clinical trials and investigators for patients enrolled in clinical trials.

#### Patient and target positioning

- Make immobilization devices that enhance reproducibility of patient setup over time so serial images can be used for quantitative treatment assessment and subsequent treatment planning.
- Incorporate interchangeable fiducials in the immobilization devices to facilitate merging the scans from two or more types of instruments, such as MRI, CT, and PET.
- Develop tools to quantitatively review localization images with field outline and anatomy contours exported from the treatment management system.
- Develop tools to quantitatively monitor daily setup correction trends for patient positioning such as from on‐board imaging or other methods.

#### Motion assessment and management

- Provide online 4D tools such as 4D CBCT capability at the treatment machine to support protocol motion management requirements.
- Provide tools to document range of motion on platforms for different imaging platforms.

#### Treatment planning considerations

- Include DICOM‐RT export in the base purchase of a TPS rather than an add‐on option with the ability to export anonymized cases to the QA centers (including image datasets, plans, structures, and dose).
- Provide standard target and structure names as provided by the QA centers or allow upload of files with the names of the structures (as defined in AAPM TG 263)
- Enable use of protocol‐specific scripts including standard target and structure names (AAPM TG 263).
- Create interfaces that import the necessary standard names, beam arrangement (if appropriate), and other information for treatment planning.
- Create the appropriate software to allow automatic anonymization with coded ID labels of patients and plans.
- Develop and make available a straightforward export of information to QA centers
- Make treatment planning systems IHE‐RO compliant
- Enable tools or scripts that can be shared and then used at the local institution to assess protocol compliance are invaluable.

Conflict of interest: Andrea Molineu is affiliated with the Imaging and Radiation Oncology Core in Houston and James Galvin is affiliated with the Imaging and Radiation Oncology Core in Philadelphia
